# Motivations and Barriers to Routine HIV Testing Among Men Who Have Sex with Men in New York City

**DOI:** 10.1007/s10461-022-03679-5

**Published:** 2022-05-10

**Authors:** Paul Kobrak, Robert H. Remien, Julie E. Myers, Paul Salcuni, Zoe Edelstein, Benjamin Tsoi, Theodorus Sandfort

**Affiliations:** 1 New York City Health Department, HIV Prevention Program, 42-09 28 Street, Long Island City, NY 11101 USA; 2 HIV Center for Clinical and Behavioral Studies, New York State Psychiatric Institute and Columbia University, New York, NY USA; 3 MetroPlus Health Plan, New York, NY USA; 4 Relevant Healthcare Technologies, New York, NY USA

**Keywords:** Men who have sex with men (MSM), HIV testing, HIV self-testing, HIV stigma, Medical care, Qualitative research

## Abstract

In-depth qualitative interviews explored the experiences and understandings of men 18–39 years old who have sex with men that could facilitate or prevent HIV testing and routine HIV testing. For many men who tested frequently, testing and routine testing were motivated by awareness of the benefit of prompt treatment; public health and provider encouragement to test periodically; responsibility towards sexual partners; and wanting to share a recent HIV-negative test result when seeking sex online. For some men, any testing was impeded by anxiety around possible HIV diagnosis that made testing a stressful occasion that required time and energy to prepare for. This anxiety was often compounded by stigma related to sex between men, having condomless sex, or having HIV. Routine testing could be further stigmatized as some men felt judged by testing providers or partners if they asked for a test or said they tested frequently. We describe efforts to promote testing and routine testing by countering fear and stigma associated with HIV and testing.

## Introduction

Frequent, routine testing and prompt diagnosis of HIV improves care outcomes and reduces onward transmission [1–8]. The U.S. Centers for Disease Control and Prevention (CDC) recommends all men who have sex with men (MSM) get tested at least annually for HIV, and suggests that “sexually active gay and bisexual men may benefit from getting an HIV test more often, perhaps every 3 to 6 months” [9–11]. The CDC has not formally recommended testing every 3 to 6 months—citing the lack of evidence of benefit or feasibility—but some local health departments, including New York City’s—have actively encouraged MSM to test with such frequency [12].

The manifest benefits of routine testing (i.e., every 3 to 6 months) have increased with the recommendation that all persons diagnosed with HIV immediately begin antiretroviral treatment [13, 14]; evidence that people who are on treatment and virally suppressed do not pass HIV to their sexual partners [7, 15–17]; and the emergence of PrEP (pre-exposure prophylaxis) as an effective prevention strategy to offer people who test HIV-negative and may be at risk of exposure to HIV [18].

How many MSM actually test as often as every 3 to 6 months? Limited data are available on the prevalence of such frequent, routine testing [19]. However, different studies have found that among U.S. MSM, 39% to 67% reported testing for HIV in the past 12 months [20–23]. While this proportion is increasing [22, 23], many MSM still test less than once a year.

Promoting routine HIV testing requires an understanding of what may encourage or inhibit *any* testing among MSM [24, 25]. Motivations to test include awareness of the benefits of HIV treatment; having HIV-related symptoms [26, 27]; having had sex without condoms or with HIV-positive partners [26, 28–30]; and feeling a sense of responsibility towards oneself, one’s partners [24, 30] or the wider community of MSM [31]. Documented barriers include the time it takes to get tested [20, 26, 29]; not thinking oneself at risk for HIV infection [26, 27, 29, 32, 33]; trusting one’s relationship partner [34]; being viewed as promiscuous for even getting an HIV test [35]; and anticipating judgment and heterosexism in healthcare settings [36]. Some men express a reluctance to test for HIV as they fear testing positive [26, 32, 35] and becoming an object of stigma from other gay men or one’s wider social circle [36–38], or because they fear medical providers might disclose their status [39].

Few studies directly address what might enable or prevent a practice of *routine* HIV testing. Hussen, et al. [40], describe a typology of testing motivations among Black MSM, distinguishing men who test: (1) regularly as part of routine self-care; (2) depending on their relationship status or sexual practices; (3) only when convenient; or (4) who avoid testing. To promote routine testing, the CDC has encouraged the routine offer of HIV testing in medical care [9]. A 2010 New York State law enacted this recommendation by requiring an annual offer of HIV testing in primary care and hospital settings, and led to modest increases in testing in clinical settings [41]. Limited research has explored how a medical provider’s approach can encourage or discourage a routine practice of HIV testing [42, 43].

We conducted this study to investigate what can motivate or limit HIV testing and frequent testing among MSM, with the larger aim of understanding how to promote routine testing. To explore these questions in depth, we interviewed a diverse sample of MSM to capture their experiences and understandings of HIV testing in their own words. This study took place in New York City (NYC), where over 1200 MSM were newly diagnosed with HIV in 2016 [44].

## Methods

We conducted in-depth, qualitative interviews with 71 cisgender men who have sex with men. With the goal of examining the range of approaches to HIV testing in a diverse group of MSM, we purposively sampled roughly equivalent numbers of men who reported testing frequently (having tested 4 or more times in the past 2 years; n = 24); less frequently (2 or 3 tests in the past 2 years; n = 25); and infrequently or never (0 or 1 tests in the past 2 years; n = 22). We recruited participants online, in-person, and through referrals from other studies, inviting all eligible recruits to be interviewed as long as slots were open in their testing-frequency category (Table 1). Recruitment online and through referrals allowed us to consider individuals’ characteristics before reaching out to them and pursue diversity in age, race/ethnicity, and testing frequency. We set a goal that at least 25% of participants identify as Latino and 25% as non-Latino Black, as members of these groups constitute 80% of newly diagnosed MSM in NYC [44]).

**Table 1:** Participants by age, race/ethnicity and HIV testing frequency

	Frequent testers		Less frequent testers		Infrequent or never testers		Study overall		
*Age* →	18–24	25–39	18–24	25–39	18–24	25–39	18–24	25–39	Sub-totals
*Race/ethnicity* ↓									
Latino	2	4	4	6	4	3	10	13	23
Non-Latino Black	3	4	2	5	1	3	6	12	18
Non-Latino White	4	3	1	2	2	4	7	9	16
Other	1	3	1	4	2	3	4	10	14
*Sub-totals*	10	14	8	17	9	13	27	44	
*Totals*	24		25		22		71		

For this analysis, eligible recruits lived in NYC; spoke English or Spanish; identified as men; and had anal sex with a man in the past 6 months. We generally limited eligibility to men who had never been diagnosed with HIV; however, to facilitate recruiting infrequent testers we included men who had been diagnosed with HIV in the past year if they had not had an HIV test in the 2 years prior to diagnosis. We limited participation to men 18 to 39 years old as this age group includes nearly 80% of newly diagnosed MSM in NYC [44]).

The interview documented participants’ experience with HIV testing through a series of open-ended questions about: their sexual practices and use of condoms and PrEP; their first and most recent tests—including what motivated them to get tested and how they felt while testing and waiting for results; their lifetime pattern of testing; situations in which they were reluctant to get tested; unpleasant testing experiences; experiences with testing during medical care; communication about testing with partners, friends and family; when and why they might get tested again; and what they thought about testing every 3 to 6 months. Many questions were broadly related to HIV testing but allowed participants to reflect on repeat or routine testing—a focus of this study. For this qualitative study, we did not attempt to quantify participants’ number of sex partners or frequency of condom use or use these characteristics to compare individual approaches to testing.

We conducted interviews from November 2015 to September 2016, in person, in English or Spanish, in settings that afforded privacy and were agreed to by the participants. Interviewers were trained by experienced qualitative researchers in the aims of study and interviewing and coding techniques. Interviews were digitally recorded, professionally transcribed, and entered into an online qualitative software program, Dedoose, to analyze content.

Following a thematic analysis approach [45], different research team members coded the content of each transcript by major theme, and another member confirmed or altered these codes, aligned the coding scheme across interviews, and identified sub-themes. As team members broadly agreed how to categorize content, we made no attempt to measure inter-coder reliability. To ensure a reliable interpretation of interview content, we emphasize direct quotations from participants and transparently report our methods and the questions we asked [46]. The first round of coding categorized content by themes derived from our question topics and research question on what may motivate or preclude routine HIV testing, including: why test; why not test; the moment of knowing one’s status; HIV testing and counseling experiences; experiences in medical care; and discussion of HIV testing and status with sex partners, friends and family. We further categorized coded content by sub-themes that emerged in the interviews. Sub-themes for “why test” included sexual risk, relationship dynamics, and testing promotion. Sub-themes for “why not test” included anxiety, stigma, and the difficulty of one’s first HIV test. We then identified patterns and compelling examples in the coded content, and reviewed whole interview transcripts to place selected quotations in the context of the participant’s experience of sex and HIV testing. In this paper, we selected and ordered these themes and related quotations to reflect how participants as a group understood and approached HIV testing and frequent testing.

Participants provided informed consent and received $50 as compensation for what was typically a one-hour interview. The Institutional Review Boards of the NYC Health Department and New York State Psychiatric Institute approved the study protocol.

### The Sample

We recruited 71 participants through a variety of sources: 20 through Grindr, Jack’d, and Adam4Adam—online sex hook-up apps and websites used by MSM; 11 from the Craigslist.com volunteer opportunities board; 7 during in-person outreach in public places and events where MSM socialize; 6 at gay bars; 1 through passive distribution of recruitment cards at an NYC Sexual Health Clinic; and the rest through referrals, including from an NYC Health Department study evaluating the HIV Home Test Giveaway [47] (16), another study of HIV self-testing (3), an HIV prevention and care clinic (3), study personnel (3), and from other participants (1). With referrals from the NYC Health Department Home Test Giveaway, we sought out MSM who had never tested prior to their home test (10 of 16), again due to difficulty recruiting men who never tested or tested infrequently.

Participants lived throughout NYC and were diverse in race/ethnicity, age, and place of birth (Table 1): 32% identified as Latino, 25% as non-Latino Black, 23% as non-Latino White, and 20% as non-Latino Asian, Middle Eastern or multiracial; 38% were 18 to 24 years old and 62% 25 to 39 years old; and 30% were born in NYC, 45% elsewhere in the U.S., and 25% in other countries. We conducted one interview in Spanish, with a recent immigrant from Latin America. Two participants had been diagnosed with HIV in the month before the interview after not having an HIV test in the prior 2 years; the other 69 reported never having received an HIV diagnosis, of whom 14 were currently taking PrEP. Below, participants are identified by pseudonyms.

## Results

### Motivations for Testing and Routine Testing

#### Possible HIV Exposure or Opportunities to Test

We asked men who had been tested for HIV if there was a specific reason why they tested—at their first, subsequent and most recent tests. Many of the men described testing after specific incidents: including having anal sex without condoms or having the condom break; not recalling details of a recent sexual episode; being sexually assaulted; or distrusting a partner’s claim to be HIV-negative after sex without condoms. Some frequent and less-frequent testers were motivated to test by an accumulation of partners or sex without condoms. Others tested as a prelude to having condomless sex with a specific partner, or “to make sure we’re both clean” (i.e., HIV-negative). A worrying symptom could also motivate testing: various participants said they had tested in response to having a flu, rash, swollen lymph nodes or even persistent allergy symptoms. Some men last tested as part of the protocol for emergency HIV post-exposure prophylaxis (PEP) or as a requirement for initiating or continuing PrEP. Many participants said they tended to get tested when it was offered in a convenient location, and a few mentioned accepting an offer of HIV testing as a way to get a gift card or free entry into a dance club or house ball—a way that some community programs encourage HIV screening. For some men, such motivations and opportunities to test resulted in a pattern of frequent testing.

#### As a Routine Practice

Some frequent testers described HIV testing as something they did periodically, less tied to specific incidents than a regular check of an important health status. For example, Jack (who reported testing 4 times in the past 2 years), called his next test “just my 6-month.” Anthony (2 tests/2 years), said of his last test,

It was just a periodical thing – get tested like once a year, just to see.

Some men expressed little hesitation to get an HIV test, even at their first test. After the first time he had anal sex, with a condom, Evan (4 tests/2 years) was eager to establish a practice of testing every 6 months.

I just wanted to do it, just to be aware, even though I knew it was going to come back negative – just to say that I was tested.

Participants who tried to test periodically included men who regularly used condoms or had little concern about being exposed to HIV. For example, Byron (1 test/2 years), who had never tested before the Home Test Giveaway, said he would try to test every 6 months,

Because it seems like the date that I could just do it. It’s not that I really go out with guys that often. It’s just always good to be checking yourself.

Some men’s testing frequency varied with their perceived risk of HIV exposure. When Evan (4 tests/2 years) moved to NYC a month before the interview, he planned to increase his testing frequency from every 6 months to every 3 months.

Just due to being more sexually active in New York... rather than being in boring Syracuse or Buffalo.

For Warren (2 tests/2 years), testing was less a routine practice than motivated by whether he thought his relationship partner had other sex partners:

It’s important for me to get tested just, I guess, as a sanity check... I don’t really follow any every-6-months or every-3-months rules... For example, if I can trust my current boyfriend, I probably won’t get tested for a long time.

#### As a Public Health Practice

Routine testing could be motivated by public-health expectations. Ryan (3 tests/2 years) understood that he *should* get tested every 6 months:

I believe that’s the length of time you should go between tests... If that’s what they say you should do, then that’s what I’m going to do.

Asked why they had last tested, some men emphasized the benefit of routine testing to ensure prompt diagnosis and treatment. Joe (2 tests/2 years) tested every year at his annual physical exam, but was not sure this frequency was sufficient:

Who knows what’s developing in your body? A year’s a really long time to just let something simmer.

From workshops on HIV prevention, Freddy (8 tests/2 years) understood that frequent, routine testing was a way to ensure quick entry into HIV treatment and suppression of the virus to an “undetected” (undetectable) level.

You should be on top of your game… When somebody does contract it, at least you can go straight to your doctor and get on the pills right away. Whether you stay undetected or whether you’re detected, as long as you keep it stable you can live with it.

Responsibility to one’s sexual partners or larger community motivated routine testing in a number of men. Jack (4 tests/2 years) said he tested every 6 months to avoid passing HIV to someone else.

If I received it and got it and I didn’t know about it and gave it to someone else, I would feel worse than me getting it. I just think about that more than anything.

Similarly, Charles (3 tests/2 years) referred to testing every 6 months as a way to “do my job.”

When I am with someone, I just want to be true with my status, in the same way I want the person to be true with his status with me. So I always do my job – keep myself healthy. I mean, do what I have to do. So I take my responsibility.

When asked what had most influenced how they approached getting tested for HIV, 2 men of color said they were motivated to avoid HIV and test frequently by the idea that gay men or Black and Latino people were at greater risk of HIV. For Steve (4 tests/2 years), who identified as Latino and Black,

Just wanting to not become a statistic... The African-American, Black and Latino community, it’s a high rate. I don’t want to be part of that rate. I want to be different, and I want to be healthy.

Charles and Steve both mentioned routine testing as a way to stay healthy, but it was unclear whether they saw testing as a way to avoid HIV or avoid disease related to HIV.

#### The Local Environment

Men who had migrated to NYC noted how the local environment could encourage testing and routine testing. Leo (2 tests/2 years) had tested twice in his native Colombia, both times as a requirement for plastic surgery. Only after coming to New York did he become aware he could *seek out* HIV testing.

The subject is everywhere.... You go on the subway, on the train, and you see posters about HIV testing. *(Translated from Spanish.)*

Similarly, Rudy (5 tests/2 years) never tested during his sexually active teenage years in small-town Texas; once he moved to New York he began to view routine HIV testing as a beneficial practice.

I started moving around people that were more in the know, not so backwoods, and they're like, ‘Yeah, you're supposed to go get tested.’

Local testing providers could also promote routine testing. After testing HIV-negative, various men had been encouraged to get another test in 6 months’ time, or given appointments to return. Such encouragement could reinforce routine testing, at least for men who had *begun* testing. Isaac (1 test/2 years), who just had his first HIV test—testing himself through the NYC Home Test Giveaway—did not consider getting another test again until discussing the idea at his study interview.

I didn’t even know there was a thing that you have to get tested [again]. I thought it just get tested and that’s it... Now that I think about it, I probably do have to get another.

#### For Recent Documentation of Not Having HIV

Another motivation for routine testing was to share evidence of a recent test when engaging other men for sex. Hugo (2 tests/2 years) said of men who present themselves online as HIV-negative,

There’s people that say, ‘Oh, I got paper that proves it.’ They’ll say, they’ll specifically say, ‘I’ve been tested 2, 3 months ago.’

P.J. (4 tests/2 years) set a rule for himself to not go 6 months without testing so he could report his recent HIV status to potential new sexual partners.

I didn’t want to be like, ‘Yeah, my status is out of date, 6 months behind.’

Online technologies have allowed potential partners to share testing histories without the need for direct discussion. Ryan (3 tests/2 years) said he had been specifically motivated to test by

People saying in their Grindr profiles when they were last tested, stuff like that. That seems to be a pretty big reinforcement that they’ve been tested a lot more recently than I have – maybe I should go get tested, too.

Byron recently got his first-ever HIV test, in part, to allow him to add a “tested at X-X date” to his profiles on hook-up apps as he had seen other men do.

If I don’t put it on, eventually somebody could ask ‘Did you get tested? Did you not get tested?’ So, it was also a big factor for me to make sure of what I have currently.

A recent test could also—after the fact—inform how you might have acquired HIV. Asked if he saw any benefit in testing every 6 months, Richie (1 test/2 years), said that if he were to test positive he could establish from whom he acquired HIV.

It’s like you narrow it down and you do the math... You know who it was.

#### Communication Among Men Motivates Some to Test

To explore whether direct conversation between men encouraged a norm of routine testing, we asked participants if they ever discussed HIV testing with sexual partners or motivated each other to test. Various men said they had asked in some way about a man’s HIV status, often using the ambiguous and stigmatizing shorthand, “Are you clean?” But few explicitly discussed their testing history with partners. One man who did so routinely, Vin (8 tests/2 years), said

I make it a habit to regularly ask about the last time a partner was tested – and what that result was – before I have sex with someone.

Another frequent tester, Michael (8 tests/2 years), was unique in having encouraged others to test regularly, saying to new partners,

‘Oh, you haven’t been tested in a year? Maybe you should get tested more frequently.’

Conversely, James (1 test/2 years), said,

I try not to go that far. You know, telling somebody what to do.

Many men said it was difficult to discuss HIV status in sexual contexts, especially with new partners (though telling a partner they take PrEP had become commonplace). Brett (1 test/2 years), who seldom used condoms, believed it would be easier to discuss HIV and testing with a relationship partner “in the middle, when we’re doing well and we’re happy.”

I feel like at the beginning of the relationship it’s going to be even harder to talk about sexual ailments or diseases or something like that... It’s often connected with shame, and in those moments the last thing you want to do is, like, ‘Oh yeah, by the way, here’s a weakness or a chink in my armor.’

Asked what that weakness might be, Brett replied “I guess the weakness would be caring too much” about whether he or a partner might have HIV or another sexually transmitted infection.

### Barriers to Testing and Routine Testing

When asked if anything had ever delayed them from getting an HIV test, common responses among men were “sheer laziness,” “not making it a priority” or not knowing “where exactly I could go.”

#### Anxiety Related to HIV Testing

Another frequent theme was the time and effort it took to get tested, and not just the time spent waiting for the test and the results. Some described an HIV test as a major undertaking that required days or weeks to prepare for. For Caleb (1 test/2 years),

I think personally I know that if I go get tested it’s going to be kind of a couple days of worry and anxiety. And frankly, depending on what I have going on, sometimes I’m just like not down to do that.

Anxiety around possibly having HIV underlaid many men’s barriers to testing and routine testing. At age 15, Amare (4 tests/2 years) started getting tested every 3 months, encouraged by staff at an after-school program for LGBTQ + youth. When interviewed at age 20, he had only had one test in the past year. Asked why he tested less often than before, Amare mentioned the mental energy involved in “thinking about all the stuff that I did.”

To put yourself through those little thoughts, the emotions, the ‘what-ifs,’ and what am I going to do if it comes out positive, and who am I going to talk to.

More than 10 study participants used the phrases “What if” or “the what-ifs” when describing HIV-testing experiences. We asked, “How did you feel while testing, or waiting for the results?” Many men recalled their mind racing, thinking about the sex they had, the possibility that a partner had HIV, or the consequences of finding out they themselves had HIV, such as having to tell partners or family or fill out “mountains of paperwork.” To Rudy (5 tests/2 years),

I don’t feel anything when I’m doing it. It’s when I’m done, that’s when in my mind I start writing out my will [laughs]... That 20 minutes lasts like a week.

Even men who saw themselves at low risk for infection could become anxious at the remote possibility of being told they had HIV. At Jason’s (0 tests/2 years) first testing experience,

I didn’t think that anything bad would happen or, like, not-so-good results would happen. But there was a small part of me that was just, like, ‘Oh, shoot, like, what if?... What if that 5 percent kicks in? What am I going to do? Who am I going to call?’

Men varied in how fearsome an HIV diagnosis would be for them. While some noted that HIV is treatable, others emphasized the continued momentousness of testing positive. To Caleb (1 test/2 years),

HIV *has* become less scary, on paper. But I think I still have some deep-seated terror of HIV that is related to before.

Anxiety around possibly having HIV could inhibit or motivate testing. Ren, age 24 (1 test/2 years), explained why he had never had an HIV test until the Home Test Giveaway:

Partially the effort, and partially not wanting to find out that result... I think it’s just quite a large sentence that you’re going to get either way, especially if you’re positive. Your life will not be the same after that. So there’s fear there, too. But not enough, apparently, to just make myself go.

In contrast, Justin (4 tests/2 years) tested frequently to *relieve* anxiety, particularly before he started taking PrEP.

If I had the time... if it was up to me, I would get tested every 2 weeks or so... Whenever I do have sex, I normally freak out about it afterwards. So, getting tested would put my mind at ease.

David (0 tests/2 years) described a shifting balance of anxieties related to HIV testing. After his first HIV test he was too afraid to return to receive his results, and only tested again years later once his fear that he may have undiagnosed HIV grew to outweigh his anxiety around testing.

I felt like the amount of risk I had exposed myself to through having sex with people I didn’t know, and sometimes unprotected sex with people I didn’t know, had reached a level that made me more anxious than knowing if I was positive.

#### The Difficulty of a First Test

Anxiety could be particularly acute at one’s first or first few HIV tests. Felix (3 tests/2 years) expressed a common theme:

The more you do it, the less terrifying it gets.

Ryan (3 tests/2 years) said he felt awkward and nervous at his first few HIV tests. But these feelings dissipated as he repeatedly received HIV-negative results. He said he now tried to test every 6 months.

It’s a mixture of me both growing up, and getting used to the fact that you just go get HIV tests... Now I know that in all likelihood it’s not going to be positive, so there’s no need to worry too much about it.

Various men who had their first-ever HIV test through the Home Test Giveaway said testing themselves at home would make it easier to get tested again. Brett (1 test/2 years) said:

It was really cool to just go through that, to get that test... It makes me want to get tested more often.

Isaac (1 test/2 years) also felt that his recent, first-ever HIV test would make it easier to test again. Before getting tested, he had grown increasingly reluctant to get tested as he had sex with multiple partners and would have been unsure with whom he was exposed were he to test positive.

Now would be easy since I know who I’m with, what I’m doing. Back then, if I was to get tested it would be like ‘Who did I get it from?’... Now it would be easier because... there’s only one partner.

Early, difficult testing experiences could impede men from ever developing a practice of routine testing. Caleb (1 test/2 years) described a stunted relationship with testing:

Maybe because some of my early ones – early testing experiences – were really stressful that I have a tortured relationship. I think if I had ever gotten in the habit of getting tested on a regular schedule, maybe I would have–. That relationship would have changed. But I never did.

Adrian (1 test/2 years), who did not get an HIV test until age 28, described his first test by drawing parallels between coming out as a gay man and possibly coming out as HIV-positive.

It was fine, it was easy, but it was nerve-wracking... It’s not so much the disease anymore, it’s also the stigma that comes attached to it still. The test is unpleasant for obviously a multitude of reasons, but it’s really unpleasant because you fear having to, I don’t know, come out all over again.

#### Shame and Stigma

Though the study questionnaire did not include the words “shame” or “stigma,” many men used these words when describing what made getting an HIV test or routine testing difficult. Asked if anything would make it hard for him to test every 3 months, Alex (6 tests/2 years) said,

The same stigma that it’s dirty if you do it, or you should feel shameful that you have to do it every 3 months... Whether you’re hooking up or in a committed relationship, I think people would still look at it as, like, ‘Wow, why are you going so often?’ Like, ‘What’s going on? Are you not being careful? How many people are you sleeping with that you have to do it every 3 months?’

Alex was among a number of men who understood their anxiety around HIV testing as a reckoning for having had sex with multiple partners or without condoms—a view Alex ascribed to having a Christian upbringing that connects sex and disease with sin and punishment.

If you’re going to get tested, is it because you were doing things you shouldn’t have? ... Like, I should have been more careful. That way, I wouldn’t have to sit here and be stressed out about it.

Explaining why he had only ever had one HIV test, Brett (1 test/2 years) repeatedly spoke of “shame” related to HIV. But if testing every 6 months became a norm, he thought testing would be easier to do and easier to talk about.

I think it’s difficult to talk about STDs and HIV and AIDS without having this fear and shame that come from our both history and just what sex is in our culture... If we all started doing it every 6 months, then it would be something where it becomes normal and not a shame-surrounded thing.

Getting an HIV test could also be a marker of homosexuality. Byron, a Latino immigrant (1 test/2 years), had recently tested for the first time. He said,

Even mentioning the whole thing, ‘Oh yeah, I got to test myself for HIV,’ would be pretty much a predisposition that you’re seeing men. So it’s kind of a taboo situation to be talking about.

Another Latino immigrant, Mateo (0 tests/2 years), who had never had an HIV test, was reluctant to get tested because he feared his name might be entered into a registry and his family would find out.

They probably already have thoughts about me not liking women as much.

#### Understandings that Can Impede Routine Testing

How other people might view routine HIV testing discouraged some men from acknowledging a recent test. Hugo (2 tests/2 years) expressed a reluctance to tell his boyfriend about his practice of testing periodically, as it could suggest he had many other sexual partners.

If he asks, then I’ll be like, ‘Look, I got tested this time,’ but I’d never be like, ‘Well, I got tested today, and this is what came out’... I don’t want him to think I might be getting tested every 6 months or every year, because I don't want him to think ‘How many people are you really going out there messing with?’ I’d rather make it seem like I’m not really messing with too many people.

Similarly, Elijah (1 test/2 years), who had never had an HIV test before the Home Test Giveaway, viewed his boyfriend’s routine testing with distrust.

He was very frequent with getting tested, which was kind of weird to me... If we were using protection, why would you feel the need? ... I honestly thought he had to have another partner at the time to feel like he needed to get tested.

Men’s own understandings of how HIV tests work could undermine the perceived benefit of routine testing. A few men doubted that testing periodically would definitively detect chronic HIV infection, believing that HIV could “pop up” after years of repeatedly testing HIV-negative. Devon (2 tests/2 years) said,

You could be like, ‘OK, I’m HIV-negative just because the scientific things shows that I’m negative.’ You know what I mean? I could be infected for so many years and it won't show up in my system for so many years and then by the time I turn around and I look at it and it finally shows up that I'm positive, I done infected so many people.

Two infrequent testers believed HIV to be so highly transmissible that they could use their relationship partners’ HIV-negative results in lieu of getting tested themselves. Adrian (1 test/2 years) said of his boyfriend,

He got tested monthly, and I just always kind of used his test as my clear.

### Motivations and Barriers to Routine Testing in Clinical Settings

Routine HIV testing in medical care has the potential to expand frequent testing. In New York State, the offer of an HIV test is required in primary and emergency care [41]. Of 71 study participants, 39 said they had been offered an HIV test during medical care and a total of 44 said they had ever tested in a medical setting (including some men who were not offered testing but asked for it).

Routine testing could depend on whether a provider offers an HIV test, as some men were reluctant to seek it out. Brett (1 test/2 years) would accept an HIV test if offered, but would not ask a doctor for a test because of “the stigma that we have as gay people.”

There was always a reason behind me going to the doctor. I was like, ‘Might as well get the STD test or HIV test.’ But I’ve never went specifically to a clinic just for that. Like, I always felt kind of embarrassed or, I don’t know, maybe some shame that would prevent me from going to the clinic specifically for that reason.

Anxiety and stigma around HIV could be acutely felt in medical settings, discouraging testing. David (0 tests/2 years) expressed a common theme:

I mean, clinical experiences aren’t always the most comfortable.

George (1 test/2 years) feared getting tested in a clinic, comparing the experience to a scene from a science-fiction movie.

Remember when E.T. is whisked off by the government, and he’s in that containment unit? And everything’s sterile and plastic and kind of freaky and really high-stress, right? To me, thinking about getting an HIV test at a clinic, that’s a picture of the emotion that goes through me.

A fear of judgment from providers contributed to men’s testing-related anxiety in clinical spaces. Justin (4 tests/2 years), who never had a test before meeting a doctor who made him feel comfortable, had been reluctant to get tested by a doctor, in part because he anticipated having to discuss his sexual practices.

Whenever I do have raw sex with a guy... I’d rather for it to turn out whatever, and just deal with it, than just go to the doctor... I didn’t want to be judged... I just didn’t want to hear that conversation... If I’m not comfortable with the doctor, I’m not going to go to them and be like, ‘I’m having sex with guys and need to get tested for this.’

Justin had recently found a doctor with whom he had rapport, and had begun testing regularly and then taking PrEP.

My doctor, she makes me feel comfortable... So I wouldn’t hesitate as much as I used to before.

Men differed in either welcoming or dreading the clinical assessments of HIV risk that often accompany testing. Vin (8 tests/2 years) said

I like giving a sexual history and talking about my level of risk... I always find that I am more willing to talk than they’re willing to ask.

Ryan preferred talking about his sex life in spaces with a predominately gay clientele. He said of getting testing and pre-test counseling at a sex party, where he was recruited for this study:

I’m more likely to be seen as an equal. And it doesn’t smell like an office... I would mind those sorts of questions from my medical provider, because historically some medical providers have been judgmental if you tell them your sexual history.

For similar reasons, Eli (6 tests/2 years) preferred an NYC Sexual Health Clinic.

It’s just easier to use the City Clinic, especially when I was down in Chelsea, due to the demographic that was down there... I fell into that demographic, so it just was a little bit more comfortable bringing a health issue to them of a gay nature.

Some men described how an affirming medical provider had encouraged them to test more. Will (3 tests/2 years), who had only one prior HIV test, was encouraged to start routine testing when he visited an NYC Sexual Health Clinic:

They just said it’s good for you to get tested at least twice a year just to make sure that everything’s running OK.

Other men reported being asked to justify why they wanted an HIV test, a question that could impede routine testing. Eli (6 tests/2 years) contrasted his last visit to a Sexual Health Clinic—when he was asked when he last tested for HIV and then immediately offered a test—with a recent visit to a private clinic where he had to explain why he wanted a test.

It’s not that I had to ask for it that made it awkward. It was just sort of like they didn’t understand why I was asking... They should have... started a dialogue of ‘Hey, are you doing this every 6 months?’... Instead of, ‘Why do you need to get checked?’… I felt like there was just, like, some judgment there.

At his last test, David (0 tests/2 years) “described myself as having a safer sex life than I had.” The person administering the test said,

‘Well, I don’t think that you’d be here getting tested if you... felt like you were really safe having sex.’ And I felt like, (a) he was right, but (b) that it was kind of inappropriate... At the time that I got the test I did have the intention of starting to get tested more regularly, and then the person... saying I wouldn’t be there unless I had had unsafe sex made me feel judged in some way... When they gave me the results, they kind of gave me a standard ‘Get tested every 3 months.’ Like, it’s good practice to get tested every 3 months. But at the same time that was kind of contradicted by the questionnaire at the beginning where I was told that unless you’re having unsafe sex basically you shouldn’t get tested.

## Discussion

In open-ended interviews, men who have sex with men in NYC described a wide range of motivations for HIV testing and routine testing, including: awareness of the benefits of routine testing and prompt treatment for HIV; encouragement from testing providers to return periodically for testing; and public health messaging that promotes the idea of routine testing. While some men were encouraged to test by a specific incident or an accumulation of sexual partners, others tested periodically as a routine check of their sexual health, as part of taking PrEP, or as a way to provide recent evidence of being HIV-negative when engaging other men online for sex.

By our study’s design, two-thirds of study participants had tested less frequently than every 6 months over the past 2 years. These men in particular—of different ages, races, and ethnicities—described experiences and understandings of HIV testing that made it difficult for them to develop or maintain a practice of routine HIV testing. A prominent theme in the interviews was how fear and anxiety around possibly having HIV continues to make HIV testing a stressful occasion, despite improvements in HIV treatment. In men’s narratives, stress related to possible HIV diagnosis could be compounded by stigma around being a man who has sex with men or sex without condoms. Previous literature finds that such stress and stigma can delay testing [36–38, 48–52]. In one quantitative study from El Salvador, men who expressed higher internalized stigma around having sex with men were less likely to ever have had an HIV test, particularly if they had experienced discrimination from medical providers or anticipated further stigma if people knew they had gotten an HIV test [53]. Stigma around having HIV could also motivate testing. In this study, participants referred to not having HIV as being “clean” or “healthy.” While such rhetoric can stigmatize people with HIV, it also appeared to motivate some men to get routine HIV testing [54, 55].

In this study, various men mentioned receiving—or anticipating—judgment or questioning from sexual partners or testing providers if they wanted an HIV test or acknowledged testing frequently [35, 53], with some feeling marked as promiscuous or irresponsible. Fear of judgment could be heightened in medical settings [56], particularly when providers required patients to describe their sexual risk or justify why they wanted to get tested. Previous research has found that stigma around HIV testing may lead patients to obscure interest in an HIV test [57], and that traditional counseling and risk assessments may discourage testing while providing limited prevention benefit [58–60]. This study’s findings support implementing a streamlined, opt-out universal offer of HIV screening [61], as HIV risk screening and risk-reduction counseling can discourage some MSM from seeking a test or even engaging in routine medical care.

Some participants preferred getting tested in places with a predominately gay clientele, often as a way to reduce the potential for having their sexuality stigmatized or misunderstood. Although various men sought out non-medical, community settings for testing, others preferred sexual health clinics that tended to serve LGBTQ + clients, as other research has found [62].

While participants described various ways stigma and discrimination related to HIV and sex between men could impede HIV testing and routine testing, they did not mention other forms of stigma or bias in the same way. Notably, men in this racially and ethnically diverse sample did not mention the experience or anticipation of racism as a barrier to getting an HIV test. In open-ended interviews, we asked broadly about unpleasant testing experiences or what could make it difficult to get an HIV test but did not specifically ask men whether race, ethnicity, poverty, or migration experiences affected their approach to HIV testing.

The growing use of PrEP—with HIV testing recommended every 3 months—may be altering collective understandings of frequent testing among MSM. We were limited in exploring PrEP’s effect on testing as few interviewees had been taking PrEP for more than a few months. PrEP use may help establish a norm of routine testing, as people must get an HIV test before initiation and then periodically while on PrEP. However, the HIV-related anxiety and stigma that made it difficult for some men to get tested might also prevent them from initiating PrEP.

Men’s narratives of HIV testing suggest ways that testing providers—including medical providers—can address barriers to testing. They can *offer* routine HIV testing to all clients to normalize testing and increase access for men who are reluctant to *ask* for a test. They can deemphasize pre-test “risk assessment” to avoid conveying the idea that people must demonstrate their actions put them at risk of HIV before they are offered testing, and instead foster an environment that invites conversation about sexual health. Providers can present HIV testing as a routine health check—and not a one-time practice—and encourage clients and patients to return for HIV testing at regular intervals, such as every 3 or 6 months. Providers can also emphasize that having HIV is a chronic, treatable condition, and emphasize options for HIV prevention, including PrEP, while avoiding any appearance of stigmatizing common sexual practices such as sex without condoms or with multiple partners.

Public health entities can address barriers to routine testing by countering stigma associated with HIV, HIV testing, and sex between men. The NYC Health Department recently shifted its messaging and social marketing in ways that aligned with preliminary study findings. Campaigns such as “Be HIV Sure”, “Play Sure” and “Listos” encouraged cisgender gay men and transgender persons to test “every 3 to 6 months” as part of a toolkit of HIV prevention options that enables a “fulfilling, worry-free sex life.” Featuring images of men coupled with other men, these campaigns aimed, in part, to make it easier for MSM to engage in HIV testing, treatment, and prevention by countering the shame associated with homosexuality and having multiple partners or sex without condoms [63]. The “Made Equal” campaign subsequently promoted the scientific finding that virally suppressed persons do not transmit HIV to their sexual partners, in part to address the fear of testing positive and reduce the stigma facing persons with HIV [64]. Recent guidance for medical providers discourages a focus on “risky behaviors” and risk assessment; promotes a non-judgmental clinical approach that welcomes MSM to disclose their sexuality; and informs clinicians that HIV testing can provoke—but also potentially relieve—anxiety [65]. To help younger men establish a practice of routine HIV testing following sexual début, pending interventions include revising NYC’s school-based health education [66] to more effectively affirm the lived experience and sexualities of LGBTQ+ youth. Public health authorities can also direct funding to ensure access to HIV testing in more appealing, non-clinical settings, including at home and at community events.

### Strengths and Limitations

In-depth qualitative interviews allowed us to capture the experiences and understandings of HIV testing of a sample of MSM, in their own words, and to place participants’ approach to HIV testing in the context of their personal experience with sex, health care, and membership in a stigmatized group. This qualitative study was not designed to represent the testing practices of all men who have sex with men in NYC or to look for correlations between patterns of sexual activity and HIV testing. Instead, we sought to describe a range of experiences with HIV testing, identify barriers that individual men face, and inform the promotion of routine HIV testing. The experiences of men in NYC may differ from places with lower incidence of HIV or less access to HIV testing.

Given our research design, this paper may emphasize barriers to testing and routine testing to a greater degree than they affect MSM in NYC. Frequent testers were easier to recruit than infrequent or never testers, suggesting that routine testing may be more common among MSM in NYC than among the men in this study. To recruit never or infrequent testers, we relied on 16 referrals from a study evaluating the NYC Home Test Giveaway [47], including 10 men who reported that they had never had an HIV test before receiving their self-test kit. Home delivery of a free test kit was an intervention that only 16 men received, and could be viewed as a source of bias as it altered these men’s experience of HIV testing. The Giveaway made it easier for the 10 men who had never previously tested to overcome their barriers to a first test. However, the perspectives of these men were invaluable in describing barriers some MSM face, including a desire to maintain privacy and to avoid clinical settings and potentially judgmental testing providers. Their experience demonstrates the important role that subsidized home self-test kits could play in facilitating HIV testing and a practice of routine testing among MSM [59], including during public health emergencies.

## Conclusion

Many MSM in this study understood HIV testing as a routine check of their sexual health that could enable prompt HIV treatment. Others tested frequently as a way to provide sexual partners with recent evidence of not having HIV. Still, some men continued to experience getting tested as stressful, often fueled by stigma related to HIV or judgment from partners or testing providers if they tested frequently. Barriers to HIV testing and routine testing include the ways testing is presented by providers, often as an outcome of an HIV risk assessment, and how testing can be understood by men, including as a kind of day of judgment for having anal sex without condoms or with multiple partners. These findings suggest that universally offering opt-out HIV testing and promoting the effectiveness of HIV treatment and PrEP can reduce remaining barriers to HIV testing and routine testing.

## Data Availability

N/A.

## Code Availability

N/A.
